# The effect of an affordable daycare program on health and economic well-being in Rajasthan, India: protocol for a cluster-randomized impact evaluation study

**DOI:** 10.1186/s12889-016-3176-9

**Published:** 2016-06-09

**Authors:** Arijit Nandi, Shannon Maloney, Parul Agarwal, Anoushaka Chandrashekar, Sam Harper

**Affiliations:** Institute for Health and Social Policy & Department of Epidemiology, Biostatistics & Occupational Health, McGill University, 1130 Pine Avenue West, Montreal, QC H3A 1A3 Canada; Institute for Financial Management and Research—Leveraging Evidence for Access and Development (IFMR-LEAD), Chennai, Tamil Nadu India

**Keywords:** Child day care centers, Childcare, Nurseries, Women’s empowerment, Socioeconomic status, Health, Cluster randomized controlled trial, India

## Abstract

**Background:**

The provision of affordable and reliable daycare services is a potentially important policy lever for empowering Indian women. Access to daycare might reduce barriers to labor force entry and generate economic opportunities for women, improve education for girls caring for younger siblings, and promote nutrition and learning among children. However, empirical evidence concerning the effects of daycare programs in low-and-middle-income countries is scarce. This cluster-randomized trial will estimate the effect of a community-based daycare program on health and economic well-being over the life-course among women and children living in rural Rajasthan, India.

**Methods:**

This three-year study takes place in rural communities from five blocks in the Udaipur District of rural Rajasthan. The intervention is the introduction of a full-time, affordable, community-based daycare program. At baseline, 3177 mothers with age eligible children living in 160 village hamlets were surveyed. After the baseline, these hamlets were randomized to the intervention or control groups and respondents will be interviewed on two more occasions. Primary social and economic outcomes include women’s economic status and economic opportunity, women’s empowerment, and children’s educational attainment. Primary health outcomes include women’s mental health, as well as children’s nutritional status.

**Discussion:**

This interdisciplinary research initiative will provide rigorous evidence concerning the effects of daycare in lower-income settings. In doing so it will address an important research gap and has the potential to inform policies for improving the daycare system in India in ways that promote health and economic well-being.

**Trial registration:**

(1) The ISRCTN clinical trial registry (ISRCTN45369145), http://www.isrctn.com/ISRCTN45369145, registered on May 16, 2016 and (2) The American Economic Association’s registry for randomized controlled trials (AEARCTR-0000774), http://www.socialscienceregistry.org/trials/774, registered on July 15, 2015

## Background

In low- and middle-income countries (LMICs), access to affordable daycare services, defined as “any type of institutional out-of-home care for children younger than five years of age” [1], is limited. India, the setting for our trial, lacks a cohesive system of reliable and affordable daycare services. Recent government efforts, including the expansion of financial assistance for crèches (nurseries where young children are cared for during the workday) in 2005–2006 under the auspices of the Rajiv Gandhi National Crèche Scheme, remain inadequate [2]. A parallel program, the Integrated Child Development Scheme (ICDS), provides meals to children through local facilities called anganwadis, and expansion of the ICDS into a daycare program has been proposed. However, anganwadis reach only about one of every six children and are marked by insufficient hours of operation, poorly trained workers, chronic staff absenteeism, and substandard facilities [3]. It is unclear how existing programs can be adapted to satisfy the demand for full-day childcare.

Women are often charged with the dual responsibilities of child rearing and time-intensive domestic work [3]. Limited access to daycare represents a potential barrier to paid employment, whether in the formal or informal labor market [4, 5]. Women who do enter the labor market by taking up paid employment often retain responsibilities for unpaid domestic work and childcare, forcing them to bring children to work, leave them home unattended, or entrust their care to older siblings [3]. The demand of caring for younger siblings is a key explanation for why adolescent girls, on average, dropout from school before boys [5]; this reduces the economic opportunities available to women directly by limiting their educational achievement and literacy and indirectly by hastening marriage and the time to first childbirth [6, 7].

**Fig. 1:**
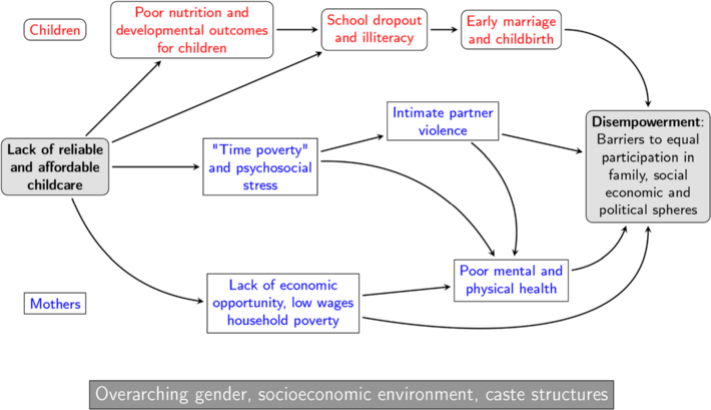
Conceptual model

The provision of affordable and reliable daycare services is a potentially important policy lever for reducing gender inequality, improving health and socioeconomic well-being, and empowering women. For mothers, access to daycare might reduce barriers to labor force entry and generate economic opportunities, which is one of the building blocks for empowerment [8]. Additionally, access to daycare might improve women’s mental health. For example, women report that they perform domestic duties out of compulsion rather than choice [9]. Access to daycare could alleviate “time poverty”, lower stress levels, and improve mental health and subjective well-being by reducing the conflicting demands on women’s time and increasing women’s autonomy. The provision of daycare may also benefit children and adolescents by removing the responsibility of caring for younger children, which limits educational opportunities. Under-nutrition and illiteracy remain challenges in many LMICs, especially India [10], and daycare programs could also improve children’s health and development outcomes through the provision of meals and learning programs. A conceptual model illustrating the potential links between lack of affordable and reliable daycare services and women’s diminished empowerment is shown in Fig. 1.

Few empirical studies have evaluated the effects of providing daycare services in LMICs. Several quasi-experimental studies have examined impacts related to women’s economic opportunities and generally report positive effects on the probability of employment and hours worked [11–16]. Scarcely any studies have examined effects on other household members, although a randomized controlled trial in rural Mozambique showed that educational attainment for older siblings improved in households where a younger child attended preschool [17]. A systematic review published in 2012 [1] concluded that the influence of daycare on child health and nutrition is unclear, although three studies reported positive effects on developmental outcomes [18–20].

There are several ways to build on extant work. With respect to internal validity, the existing evidence draws heavily from non-experimental studies that are vulnerable to bias from unmeasured confounding. Appropriately designed quasi-experiments, for example by using instrumental variables, can be useful, but they rely on important assumptions that cannot be verified. Moreover, prior research utilizing instrumental variables [14] has reported effects on nutritional outcomes that were of a biologically implausible magnitude [1]. With respect to external validity, prior work is derived almost entirely from Latin American countries and may not be generalizable to other regions, including South Asia.

The demand to facilitate women’s labor force participation by providing access to affordable daycare services will continue to increase if India, as well as other LMICs, are to promote inclusive economic growth. However, there is presently a lack of credible research for constructing evidence-based policies for improving access to daycare in India in ways that benefit the health and socioeconomic well-being of women and children. This paper presents the protocol for a cluster-randomized trial, named the *Uttam Unnati,* or “great progress” study, which aims to address this research gap by evaluating the effect of a community-based daycare program on health and socioeconomic status among women and children living in rural Rajasthan, India.

## Methods

### Study design

**Fig. 2:**
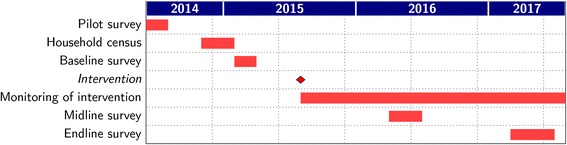
Study design and research timeline

This is a three-year cluster-randomized trial that examines the effects of introducing an affordable daycare program on health and economic well-being over the life-course. Briefly, a census was completed in late 2014, followed by a baseline survey in early 2015 among 3177 mothers with a child between one and six years of age living in 160 village hamlets from five blocks in the Udaipur district of Rajasthan. In the fall of 2015, Seva Mandir, a local non-governmental development organization that operates daycare programs in other areas in the Udaipur district, established daycares, called *balwadis*, in 80 randomly selected hamlets. The remaining 80 hamlets serve as control groups that are ineligible to receive a balwadi until after September 2017, by which point mothers will have been re-interviewed twice, in early 2016 and again in early 2017. The study timeline is shown in Fig. 2.

### Sample selection

#### Power calculations

Power calculations were used to determine the sample size needed to detect an impact of our intervention on one of the study’s primary outcomes, women’s labor force participation. Given our basic cluster-randomized framework, with clusters specified at the level of the hamlet, the power analysis was based on the following parameters: the significance level, 1 − *α*, where *α* represents the probability of a type I error; the sample size per cluster, *n*; the number of clusters, *J*; the fraction allocated to treatment versus control, *P*; and the between and within-cluster variances, *τ*^2^ and *σ*^2^, respectively. Some of these parameters were considered fixed. In particular, we assumed a fixed number of 160 clusters given the finite number of treatment naïve hamlets available in our study area. We assumed that the proportion of women in the labor force was 30 % (*variance* = 0.3 * (1 − 0.3) = 0.21) based on pilot data that we had collected in areas lacking access to the balwadi program. We varied other parameters, specifically the sample size per cluster, in order to estimate the sample size necessary for achieving a power, (1 − *κ*), of 80 % for estimating a feasible and relevant minimum detectable effect size (MDE) in the presence of clustering, measured by the intra-cluster correlation (ICC). The MDE is given by [21]:

$$ MDE=\left({t}_{\left(1-\kappa \right)}-{t}_{\alpha /2}\right)\times \sqrt{\frac{1}{P\left(1-P\right)}}\sqrt{\frac{n{\tau}^2+{\sigma}^2}{nJ}} $$

For degrees of clustering typical in social science surveys (between 0.01 and 0.05) [22], we determined that a sample size of 20 individuals per 160 clusters was adequate to provide 80 % power to detect MDEs that seemed feasible and relevant (5 to 7 percentage point differences in labor force participation rates). We consider this sample size to be conservative because it assumes no baseline covariates. We have collected information in the baseline survey on pre-treatment covariates (including primary outcomes) and plan to include these covariates in our analysis of our outcomes, which should improve our precision [21].

#### Participants and village hamlets

**Fig. 3:**
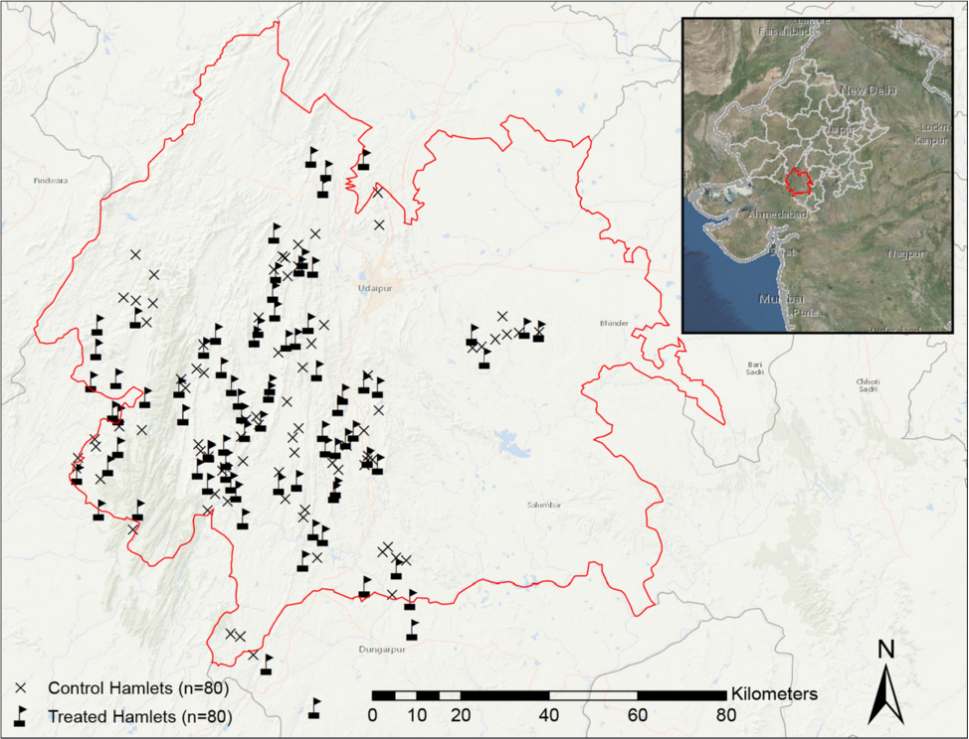
Locations of study hamlets in Udaipur District, Rajasthan, India

In December 2014 and early January 2015, 160 hamlets were selected from five blocks (i.e., Badgaon, Girwa, Jhadol, Kherwara, Kotra) in the Udaipur District where Seva Mandir had not previously established balwadis (Fig. 3). These hamlets satisfied five criteria determined *a priori*, specifically: (1) no readily accessible daycare within 1.5 kilometers to reduce the potential for contamination effects; (2) a minimum number of children (≥25) in the appropriate age range in the hamlet to ensure adequate demand; (3) an existing structure suitable for a daycare; (4) a qualified woman, living in the study hamlet or nearby, to operate the daycare; and (5) adequate demand from the village council (*Panchayat)* for a new daycare.

### Survey procedures and participants

#### Survey procedures

The survey questionnaire was translated from English to the localized Hindi language by a professional translator. The translator was instructed to retain the meaning of the questions as they were written in English, but to translate them for the local Hindi speaking audience. Once the translation was complete, a project staff member with knowledge of the local dialect reviewed the questionnaire to ensure that the Hindi version kept the original intent of the questions.

The survey software was designed on SQL by the Institute for Financial Management and Research—Leveraging Evidence for Access and Development (IFMR-LEAD) software team. The final software format was designed through an iterative process. The research associate gave an a initial set of written instructions to the software team, then tested each version of the electronic questionnaire and gave feedback to the team until all errors were corrected. The instructions included rules to reduce surveyor error in data entry, including: automatic skips across appropriate questions, acceptable ranges for relevant variables, flags or error messages when an invalid input has been placed, etc. The final version of the survey included a fully functioning set of rules and skips to minimize error in the field.

We tested the face validity of the questionnaire by consulting individuals with local expertise and added, deleted, and revised existing indicators. In mid-2014 we pilot tested the questionnaire in a convenience sample of approximately 200 women from hamlets outside of our sampling frame. The questionnaire was modified based on the results of the pilot study.

We took multiple steps to correct and minimize potential for survey error. The survey supervisor observed each surveyor for one survey each day, monitoring for correctness in asking survey questions, providing necessary clarifications, and responding to respondent questions. A random set of 10 % of completed surveys was selected for back-checks, done to verify that each surveyor recorded the appropriate responses. To do this, the back-check team is given a list of questions asked in the survey that are unlikely to change over a short time period, like the number of children, respondent’s marital and educational status and employment history. The back-check team independently re-interviewed the respondents and entered their responses into a software program designed to test these questions for identical responses. When a discrepancy is found between the original survey response and the back-check response, a third party was sent to verify the response. The response found to be correct by the third party is taken as the final response.

#### Study participants

In late 2014, we completed a household census in each of the 160 hamlets to confirm the eligibility of the hamlet, enumerate the population, and identify potential respondents for inclusion. Eligible households were those with at least one mother (biological or guardian) with a child between one and six years of age. At this time, the respondent was considered eligible if they responded to the question “Do you have any children between one to six years of age” with a yes. Based on this, the total number of eligible households (*n* = 3899) was similar to our desired sample size. From this list, we randomly selected one eligible respondent from each eligible household to complete a baseline survey. At the time of the baseline survey, we developed a more rigorous approach for measuring the age of the respondent’s children, which included mapping dates to the respondent’s life history, local events and major holidays. This more rigorous method revealed that several “eligible” children were actually older or younger than our target population. A total of 343 respondents were labeled as ineligible due to not having a child between one to six years of age and these households were dropped from our sample.

Potential respondents were given the opportunity to refuse or consent to participation in the study. After describing the study objectives, procedures, potential risks, potential benefits, voluntary nature, confidentiality and privacy protections, and compensation, each eligible respondent was asked if they would consent to participate. This was done in written form for respondents who could read and write and orally for those who could not. Each respondent who agreed to participate received a blanket, valued at 100 rupees (Rs.), as compensation at the completion of the survey interview.

After accounting for the households that refused consent, could not be successfully reached after three visits, had migrated to another location, were ineligible or were deemed unable to respond due to any kind of illness or handicap, a total of 3177 respondents were included in the final sample (response rate = 89.0 %). The mean completion time for the baseline survey was 50 min. Follow-up surveys of baseline participants are scheduled for early 2016 (approximately six to eight months after the intervention started) and again in early 2017.

### Randomization and intervention

#### Randomization

We used a stratified randomization procedure to randomly assign the 160 hamlets to treatment or control. We stratified by block (*n* = 5) to prevent variations in the distributions of blocks across treatment groups (e.g., if women in treated hamlets were more likely to reside in blocks with more economic opportunities). Randomization was performed at McGill University by an investigator (SH) using a de-identified listing without block or hamlet names. Each hamlet was randomized to either the treatment or control within blocks using a random number generator in Stata software. Because four of the five blocks contained an odd number of hamlets, the randomization was done so that two of the four blocks would have an additional treated hamlet and the remaining two would have an additional control hamlet (so the result is 80 treated hamlets). The de-identified listing, now with a treatment variable, was merged with identifying information, including block and hamlet names, by a separate investigator (AN) and conveyed to Seva Mandir for implementation. Table 1 shows the distribution of socio-demographic characteristics at baseline for the total, treated, and control groups. In general, baseline characteristics were balanced between the treatment and control arms of the trial, in both individual and cluster-level analyses.

**Table 1:** Baseline characteristics for the total sample and stratified by treatment arm, presented at the individual (*n* = 3177) and cluster (*n* = 160) levels

	Individual-level analysis								
	Total sample			Control hamlets			Treated hamlets		
Variable^a^	No.	Mean	SD	No.	Mean	SD	No.	Mean	SD
Age (years)	3169	29.87	6.86	1517	29.87	6.90	1652	29.86	6.83
Any schooling	3175	0.26	0.44	1519	0.27	0.44	1656	0.25	0.43
Married	3177	0.98	0.12	1521	0.99	0.11	1656	0.98	0.14
Age married (years)	3070	17.47	2.85	1467	17.49	2.70	1603	17.44	2.98
No. sons	3177	1.62	1.16	1521	1.62	1.16	1656	1.61	1.16
No. daughters	3177	1.65	1.27	1521	1.60	1.25	1656	1.69	1.28
Hindu religion	3175	0.72	0.45	1519	0.73	0.44	1656	0.72	0.45
Worked in past 7 d	3177	0.59	0.49	1521	0.59	0.49	1656	0.59	0.49
Worked in past 12 mo	3177	0.95	0.22	1521	0.93	0.25	1656	0.96	0.18
Paid cash for work	3016	0.09	0.28	1420	0.09	0.29	1596	0.09	0.28
Days childcare prevents work	3016	1.53	4.66	1420	1.64	4.97	1596	1.42	4.36
Below poverty line	3168	0.50	0.50	1517	0.51	0.50	1651	0.50	0.50
	Cluster-level comparison								
	Total sample			Control hamlets			Tread hamlets		
	No.	Mean	SD	No.	Mean	SD	No.	Mean	SD
Age (years)	160	29.89	1.93	80	29.84	2.14	80	29.93	1.70
Any schooling	160	0.27	0.17	80	0.28	0.18	80	0.26	0.16
Married	160	0.98	0.03	80	0.99	0.03	80	0.98	0.04
Age married (years)	160	17.50	0.74	80	17.49	0.73	80	17.51	0.75
No. sons	160	1.60	0.33	80	1.61	0.34	80	1.58	0.32
No. daughters	160	1.65	0.34	80	1.59	0.36	80	1.70	0.31
Hindu religion	160	0.72	0.15	80	0.74	0.17	80	0.71	0.14
Worked in past 7 d	160	0.59	0.19	80	0.60	0.19	80	0.58	0.18
Worked in past 12 mo	160	0.95	0.07	80	0.94	0.08	80	0.96	0.06
Paid cash for work	160	0.09	0.10	80	0.09	0.10	80	0.09	0.09
Days childcare prevents work	160	1.60	1.67	80	1.78	1.88	80	1.41	1.42
Below poverty line	160	0.51	0.18	80	0.52	0.19	80	0.50	0.17

^a^ Variables are: age in years (age); whether the respondent ever attended school (any schooling) or not; whether the respondent was married (married) or not married, including widowed, divorced, separated, or living together married; the age at marriage, if married (age married); the number of sons under age 18 y who live at home (no. sons); the number of daughters under age 18 y who live at home (no. daughters); Hindu religion (Hindu) or other, including other religion, no religion, or unknown; whether the respondent worked in the past seven days (worked in past 7 d) or not; whether the respondent worked in the past 12 months (worked in past 12 mo) or not; whether respondents who reported working in the past 12 months were paid in cash (paid cash for work) or not; the number of days in the past month that respondents who reported working in the past year were unable to work because of childcare (days childcare prevents work); and whether the household has a Below the Poverty Line card (below poverty line)

#### Intervention

The intervention is the introduction of full time, affordable daycare centers in 80 treated hamlets in areas where they are not yet available. Each of the balwadis provides child-care, nutritious food and supplements, basic medicines, and preschool education to children one to six years old. The balwadi program also aims to increase immunization coverage of children by maintaining immunization records and following-up with parents and government nurses. Balwadis are operated by local women, called *sanchalikas*, who are hired and trained by Seva Mandir. Sanchalikas receive approximately 20 days of training each year regarding their roles and responsibilities. The sanchalikas meet with children’s families on a quarterly basis to discuss their child’s progress. The implementation of daycare programs in treatment villages was accompanied by a household marketing campaign to encourage sustained enrollment. Take-up rates of the intervention will be available after the mid-line survey is fielded.

#### Blinding

Due to the nature of the intervention it was not possible to blind study personnel or participants after the implementation of the intervention. However, we did conceal the allocation of hamlets to treatment or control status until after the baseline survey in order to minimize opportunities for bias in recruitment of participants and the baseline survey.

#### Monitoring of the intervention

We hired two field workers to conduct monthly site visits to each daycare in the treatment arm. Each month, these study monitors visit all balwadis in random order. During the visit, they verify that the balwadi is operating in the correct location, care is provided by a sanchalika, the balwadi structure is adequate, food is provided, the monitoring systems are operational; the monitors also collect other broad level indicators that the intervention is proceeding as planned.

General records for each balwadi maintained by Seva Mandir will be used to measure the average number of children attending the balwadi each month relative to the total enrollment, and the total number of days the balwadi was operating each month vis-à-vis the number of days it should have been open. The daily operation of each balwadi and attendance of sanchalikas is being assessed using a camera monitoring system. This system, which has been demonstrated to reduce teacher absenteeism [23], requires sanchalikas to take three self-timed photos each day (9:30 am to 10:00 am at opening, a second after 2.5 h, and a third after 6 h). These digital photos are then used to evaluate the number of days when the balwadi was run for at least 6 h, considered a full day of operation. The sanchalika’s payment is based on their attendance. Sanchalikas receive a monthly salary of Rs. 2,275 monthly if they are present for at least 21 days in a month. Each additional full day is incentivized with a bonus of Rs. 400 up to a maximum of Rs. 5,875 per month. Each day missed compared to the 21-day benchmark results in a penalty of Rs. 175 if the balwadi operated for more than ten days in that month and Rs. 50 if the balwadi operated for ten or less days in that month. Additionally, we are measuring how frequently each balwadi is visited by community members; Seva Mandir requires that each balwadi is visited by a delegate from the specific block level every three months, by a delegate from the local zone once a month, and by a member of the village development committee once a month. Records maintained by the sanchalika are being used to measure the materials received at each balwadi, including educational materials, medicines, toys, and utensils.

### Measures

#### Outcome measures

Our primary endpoint is women’s empowerment. We adopted the conceptual approach of Kabeer [8, 9, 24], which incorporates both the personal and political dimensions of empowerment, and developed indicators that captured “women’s sense of self-worth and identity, their willingness to question their own subordinate status, their control over their own lives, and their voice and influence within the family” [8]. We constructed indicators of empowerment, adapted from the Indian National Family Health Survey [25] whenever possible to facilitate comparability, which encompassed four domains: (1) decision making within the family and control over income (e.g., who decides how the money you earn will be used: mainly you, mainly your husband, or you and your husband jointly?); (2) freedom of movement in the public domain (e.g., are you usually permitted to go to the following places—for example, a market within the village—to buy things: on your own, only if someone accompanies you, or not at all?); (3) participation in community and public life (e.g., are you a member of any type of association, group or club which holds regular meetings?); (4) and views and attitudes on critical gender issues (e.g., please tell me if you agree or disagree with each statement: A married woman should be allowed to work outside the home if she wants to). We plan to test the reliability of empowerment measures during the second survey wave.

We will also measure the primary mechanisms hypothesized to link access to daycare to women’s empowerment, including time use, economic opportunity, economic status, and mental health and well-being.

Use of time was measured using a structured questionnaire, adapted from a study by Beaman et al. (2012) [26], that asked respondents whether they spent any time in the past 24 h on specific activities (e.g., gathering fuel or firewood), how much time they spent on each activity, and whether this amount reflected the usual amount of time spent on the activity. The questionnaire also asks whether respondents were paid in cash or in-kind for the activities they engaged in.

We asked about employment experiences, including whether respondents work, their occupation, the type of work, the quantity of work, whether they are paid for their work in cash or in-kind, and what they do with their children while working. Respondents reported the household income received in the past 12 months from various categories (e.g., agricultural income, business income, rents, remittances, government payments). Household wealth is measured using a series of questions about ownership of specific assets (e.g., telephone, bicycle, radio), environmental conditions (e.g., type of water source, sanitation facilities), and housing characteristics, (e.g., number of rooms, materials used for housing construction). Additionally, respondents are asked about savings accounts held by household members, including for each account the type of account, its purpose, the total value, and whether the respondent can use the account to make purchases.

Symptoms of common mental disorders (CMD) are being assessed using the 12-item General Health Questionnaire (GHQ-12) developed by Goldberg [27]. The GHQ-12 produces results that are similar to the longer version of the GHQ and has been found to be a valid screening instrument for CMD in diverse settings [28]. To the best of our knowledge, validation studies have not been conducted among women living in Rajasthan. However, the GHQ-12 demonstrated high sensitivity and specificity in a validation study conducted in Goa, India using a cutoff score of 5/6 [29], a sample of primary care patients in Tamil Nadu using a cutoff score of 2/3 [30], and a sample of ethnic Indian women living in the United Kingdom using a cutoff score of 2/3 [31]. We used the Hindi version of the GHQ-12 translated by Gautam et al. [32].

We asked mothers about the health of their children under the age of six. We asked about their child’s immunization coverage, including Bacillus Calmette-Guérin (BCG), Diptheria-Tetanus-Pertussis (DTP), hepatitis, measles, and polio vaccines. We also asked about the occurrence of specific symptoms over the past month, including fever, persistent cough, diarrhea, broken bones, cuts, or burns. Additionally, we measured child length/height and weight using standardized techniques and this information will be used to derive children’s length/height-for-age, weight-for-age, weight-for-length/height, and body mass index (BMI)-for-age using the World Health Organization Child Growth Standards [33].

#### Other covariates

Other covariates included socio-demographic characteristics, including respondent’s age, educational attainment, religion, caste, and marital status. We asked the respondent to report the following information for all children in the household who were less than eighteen years of age: literacy, enrollment in school, time spent in school in the prior year, highest level of education completed. For children not enrolled in school we asked about why they were not in school and their main occupation. Additionally, we asked about the husband of married respondents, including their age, educational attainment, and occupation.

### Statistical analyses

Descriptive statistics, including comparisons of means and proportions, were used to compare socio-demographic characteristics between the treatment and control arms (Table 1). We will conduct intention-to-treat analyses assuming that respondents complied with their initial treatment assignments. We will estimate the impact of the treatment, access to affordable daycare, on primary endpoints with marginal linear and log-linear regression models using generalize estimating equations to account for clustering at the hamlet level [34]. Multivariable regression will be used to control for block, which was used to stratify the randomization, and pre-treatment values of endpoints, in order to improve the precision of our estimates. Results will be reported on the absolute and relative scales as risk differences and risk ratios with their respective 95 % confidence intervals. Results will be reported following the CONSORT statements for randomized trials and cluster randomized trials [35, 36].

### Trial status

At the time of submission the intervention had been introduced in treatment areas and data collection as part of the mid-line survey was being planned.

## Discussion

This paper provides the protocol for a cluster-randomized evaluation of the impact of access to affordable daycare on social, economic, and health outcomes of rural Indian women and their children. Prior evidence of daycare impacts on women has been limited by non-randomized designs, and outcomes have generally focused on children rather than mothers. Moreover, most prior research has been conducted in Latin America.

We are monitoring and attempting to mitigate threats to the internal validity of our study, which include partial compliance, attrition, and spillovers. First, partial compliance could occur if hamlets assigned to the treatment arm did not open a daycare center or closed a daycare center before the end of the study period; this would tend to reduce the difference in treatment receipt between treated and control clusters and dilute the true treatment effect. A daycare center might close in a treated hamlet if a government anganwadi were to open in the same hamlet (Seva Mandir does not operate balwadis in hamlets with a government anganwadi). Additionally, a center might close if a sanchalika became unable to operate the center and a suitable replacement could not be identified. Seva Mandir has worked in partnership with the community in the Udaipur District since 1968 and study hamlets were restricted to areas with adequate demand expressed by village councils; thus, we do not anticipate substantial non-compliance in our treatment arm. Treatment effects might also be diluted if government anganwadis are established in control hamlets. Second, attrition could occur in our study if respondents are lost to follow-up during the study period. We are attempting to prevent selection bias by minimizing losses to follow-up; strategies include making at least three visits to respondents’ homes to conduct interviews, collecting detailed address and telephone information to track individuals over time for follow-up interviews, and providing an incentive to participate in the study. Given the relatively short time frame for our study we do not expect a substantial rate of attrition. Third, spillovers could occur if, for example, respondents of control villages enrolled their children in balwadis in treatment villages. In order to reduce potential spillovers, we utilized a cluster-level design and did not select control hamlets within a buffer of approximately 1.5 kilometers from a treated hamlet. Moreover, hamlets in the region are typically dispersed and daily travel between hamlets is relatively rare. Seva Mandir’s prior work suggests that the risk of women in a neighboring hamlet taking up day-care in a treated hamlet is low.

Rajasthan is among India’s poorer states and the results of our study may better generalize to other low-income contexts. However, there may be limitations to our study in terms of external validity and potential scale-up. Our study is being conducted in selected areas that have adequate demand to sustain a daycare center. Additionally, we are evaluating a model of daycare designed by a non-governmental organization that has been operating in the region for many years. Whether results concerning the impact of the balwadi program can be generalized to other rural areas lacking affordable daycare service within India, or to different daycare models, is unclear.

Caveats considered, there are several strengths to our study. First, randomization provides rigorous evaluation by ensuring that treatment and control groups are similar with respect to measured and unmeasured characteristics. Second, randomization at the hamlet-level limits the potential for spillover or contamination effects. Third, the extent to which the costs of day-care programs are offset by benefits is unknown and we will perform a prospective cost-effectiveness analysis to increase the policy relevance of our findings.

Cost-effective interventions to improve women’s empowerment are urgently needed, especially in regions where existing indicators suggest that women’s empowerment is low. Our cluster-level design, described in this study protocol, will help to improve the quality of evidence on whether providing community-run full-time daycare may improve economic opportunities and empowerment among rural women.

## Abbreviations

BCG, Bacillus Calmette-Guérin; BMI, body mass index; CMD, common mental disorders; DTP, Diptheria-Tetanus-Pertussis; GHQ-12, 12-item General Health Questionnaire; ICDS, Integrated Child Development Scheme; IFMR-LEAD, Institute for Financial Management and Research—Leveraging Evidence for Access and Development; LMICs, low- and middle-income countries; MDE, minimum detectable effect size
